# Advances in the Understanding of the Complex Role of Venous Sinus Stenosis in Idiopathic Intracranial Hypertension

**DOI:** 10.1002/jmri.28177

**Published:** 2022-03-31

**Authors:** Kexin Zhao, Wenjing Gu, Chunmei Liu, Derui Kong, Chong Zheng, Wei Chen, Xuewei Li, Yuchen Liang, Hongwei Zhou

**Affiliations:** ^1^ Department of Radiology The First Hospital of Jilin University Changchun China; ^2^ Department of Otorlaryngology The First Hospital of Jilin University Changchun China; ^3^ Department of Gynecology Changchun Obstetrics Gynecology Hospital Changchun China

**Keywords:** idiopathic intracranial hypertension, intracranial pressure, extrinsic factors, intrinsic factors, pathogenesis

## Abstract

Idiopathic intracranial hypertension (IIH) is a disorder characterized by elevated intracranial pressure (ICP) that predominantly affects young obese women. IIH is a diagnosis of exclusion. That is, if increased ICP is suspected, magnetic resonance imaging and magnetic resonance venography of the brain are recommended to exclude secondary causes. Imaging findings, such as empty sella, orbital findings, meningocele, and encephalocele, are not diagnostic of ICP, nor does their absence exclude ICP either. Therefore, venous manometry is recommended as the gold standard for evaluation, regardless of previous anatomic imaging results. Venous manometry is an invasive examination that is frequently applied to derive physiologic information concerning the nature of the pressure gradient. However, the pathogenesis of IIH has not been fully elucidated. The presence of venous sinus stenosis in a subset of patients has provided some support for the potential mechanisms underlying this condition. Hence, this review provides an up‐to‐date discussion on the potential pathogenic mechanisms of IIH with a special focus on venous sinus stenosis.

**Level of Evidence:**

1

**Technical Efficacy Stage:**

2

Idiopathic intracranial hypertension (IIH), formerly termed pseudotumor cerebri or benign intracranial hypertension, is characterized by elevated intracranial pressure (ICP) with no identifiable causes. It most often occurs in young, obese women of childbearing age.^1^ The clinical presentation of this disorder is highly variable but may include headaches, visual loss, diplopia, and pulsatile tinnitus.^2^ Headache and visual deterioration are the main symptoms of IIH. Headache may substantially lower the patient's quality of life and visual alterations may be irreversible due to papilledema, a condition in which increased ICP causes swelling of the optic nerve. Papilledema is encountered in almost all patients with IIH and is a good indicator of increased cerebrospinal fluid (CSF) pressure.^3^ Therefore, an accurate diagnosis of IIH informed by clinical and imaging studies is essential.^4^, ^5^

The diagnosis of IIH, which is based on the modified Dandy criteria, requires papilledema, normal CSF components with elevated CSF opening pressure and no structural causes of increased ICP on computed tomography or magnetic resonance imaging (MRI).^6^ To improve the targeted treatment of IIH, it is essential to understand the mechanisms involved and clarify its pathogenesis. Many treatment options or recommendations are driven by these theories, including those related to abnormal CSF dynamics, such as excessive secretion, reduced drainage, or both, and pressure differentials within the venous sinus system, such as venous sinus stenosis.^7^ The purpose of this review is to explore the possible pathogenic mechanisms, clarify the increasing role of imaging methods in the diagnosis of IIH, and analyze the prospects of venous sinus stenting for IIH management. According to the current state of knowledge, the most important component of the potential pathogenic mechanism of IIH is venous sinus stenosis, which provides vital theoretical support for the existence of this disease.

In this review, we aimed to evaluate the importance of venous sinus stenosis within IIH through imaging methods as well as to provide answers to the following three questions: Is there a direct relationship between the degree of venous sinus stenosis and elevation of ICP? Is there a causal relationship between venous sinus stenosis and IIH? Is venous sinus stenosis mainly caused by extrinsic or intrinsic factors? This review provides an updated, comprehensive review of IIH, including the latest and most detailed descriptions of its pathogenic mechanisms, imaging findings, and management strategies.

## Pathogenic Mechanisms

Current research supports the hypothesis that IIH is stemmed from a neurovascular etiology, including enhanced CSF production by the choroid plexus, decreased CSF drainage across the arachnoid granulations (AGs) or lymphatics, and increased venous sinus pressure.^8^ The Monro–Kellie doctrine points out that the sum of the volumes of the brain, CSF, and intracranial blood is constant and in a state of volume balance. Specifically, an increase in one of them will inevitably lead to a decrease in at least one of the remaining two. This hypothesis provides important theoretical support for the etiology of IIH.^9^ In addition, metabolic or hormonal factors may play a role in the pathogenesis of obesity, which is considered a risk factor due to the prevalence of obesity in the IIH population.^10^ In a multicenter case–control study, a high body mass index was found to be associated with an increased risk of IIH.^11^ Moreover, central obesity may increase intra‐abdominal pressure, elevate the diaphragm, increase central venous pressure, and trigger the transmission of signals to the cerebral venous system to reduce the absorption of CSF.^12^ However, the causality of these associations has not been evaluated to date. In the following sections, we will elaborate on the various pathogenic processes plausibly underlying IIH with a special focus on venous sinus stenosis.

### CSF Hypersecretion

The nervous system normally contains approximately 140 mL of CSF, a volume that replenishes three to four times a day.^13^ The choroid plexus is the main tissue responsible for CSF secretion, generating approximately two‐thirds of the total volume of the CSF, with the rest arising from extrachoroidal sources, such as the ependyma and possibly the blood–brain barrier.^14^, ^15^ Abnormalities of the choroid plexus may lead to hypersecretion.

### CSF Outflow Obstruction

**FIGURE 1:**
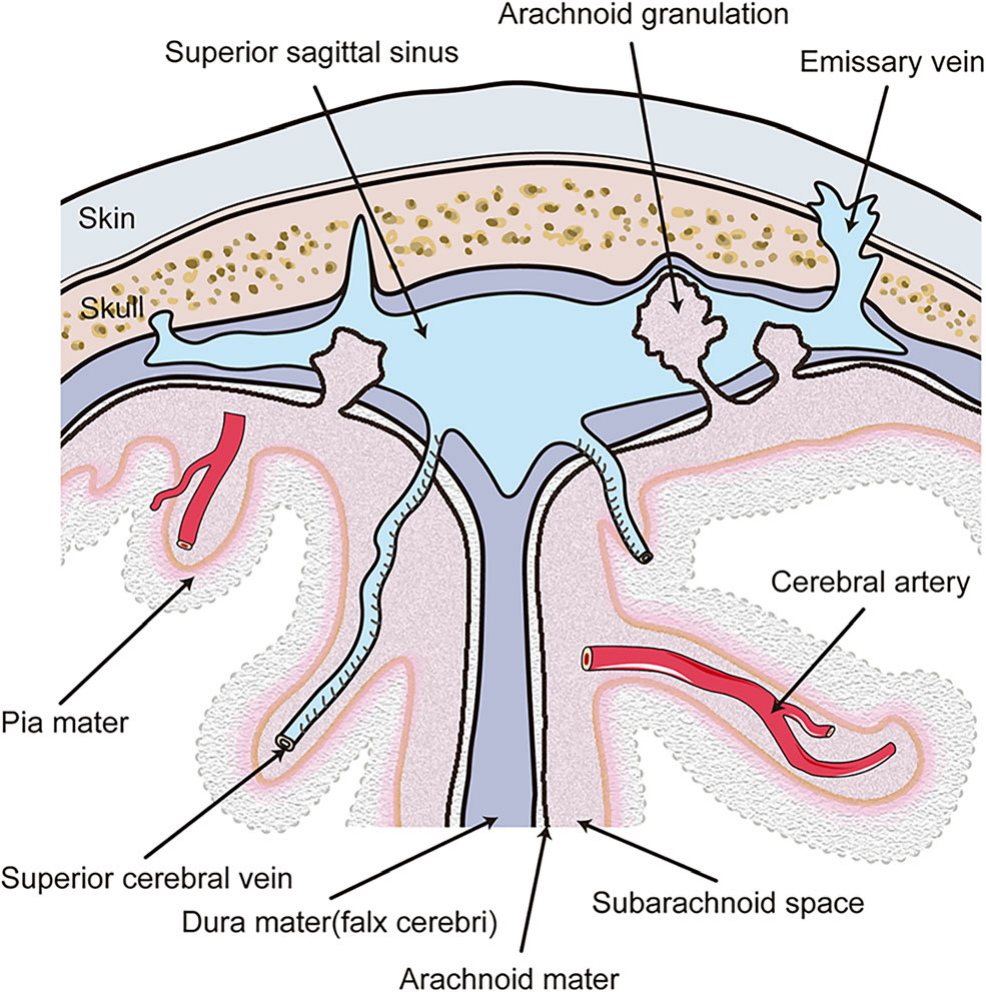
Anatomy of arachnoid granulations. Arachnoid granulations are protrusions of arachnoid through the dura mater which usually detected randomly in the dural venous sinus. These small structures are thought to provide cerebrospinal fluid drainage from the subarachnoid space into the bloodstream.

It is generally believed that drainage of the CSF is carried out through AGs (Fig. 1). AGs transfer CSF from the subarachnoid space into the systemic circulation. This process depends on the pressure gradient, such that elevations in cerebral venous pressure lead to impaired CSF drainage. The lymphatic system is another CSF outflow system that connects the cerebrovascular and CSF circulation and plays a role in IIH. More specifically, this is a glial‐dependent perivascular network composed of CSF and interstitial fluid that removes metabolic waste and connects to the venous system.^16^ Studies have shown that the expansion of the brain and interstitium caused by lymphatic dysfunction may be responsible for the extrinsic venous sinus stenosis of IIH. Although clear confirmation of this hypothesis has yet to be obtained, it is apparent that the dysfunctions of the various CSF drainage modes in IIH are neatly intertwined.^17^

### Venous Sinus Stenosis

**FIGURE 2:**
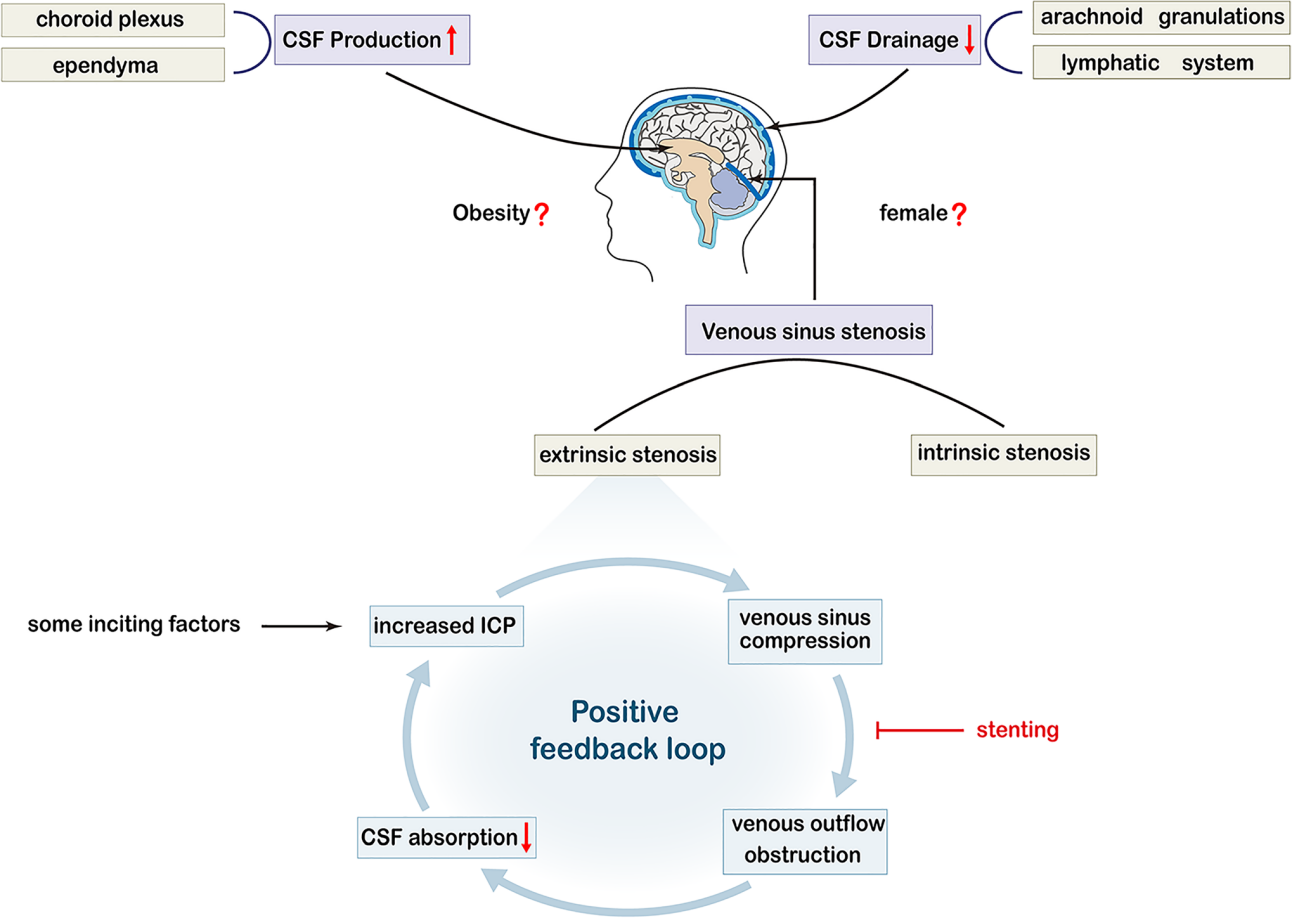
Schematic illustration of the possible pathophysiological mechanisms of idiopathic intracranial hypertension (IIH). Three primary mechanisms have been suggested to lead to IIH: enhanced cerebrospinal fluid (CSF) production at the choroid plexus or ependymal cells, decreased CSF drainage across the arachnoid granulations or lymphatic system, and venous sinus stenosis, which is the most important part of IIH and can be intrinsic or extrinsic. Some inciting factors such as weight gain and hormonal changes can cause an elevation in intracranial pressure (ICP), compressing the collapsible transverse sinus, causing extrinsic compression, blocking the venous outflow, leading to further venous hypertension, reducing the absorption of CSF, causing a consequent further elevation in ICP, and thus perpetuating the entire cycle. This positive feedback loop is referred to as the Starling‐like resistor. This vicious circle can be ended by some treatments, such as venous stenting. In addition, obesity and female sex are known predisposing risk factors for IIH development and may directly affect CSF dynamics.

Venous sinus stenosis is increasingly recognized as a crucial component of the pathogenesis underlying increases in ICP and is considered a vital part of IIH. Elder et al^18^ reported that the incidence of transverse sinus stenosis in patients with IIH ranges from 10% to 90%, while the incidence in the general population is 6.8%. It was not until the end of the 20th century, with the exploration of morphological abnormalities thanks to the advancement of imaging, that venous sinus stenosis once again became the focus of IIH research and medical practice.^19^ Venous sinus stenosis typically leads to the collapse or flattening of the expected contour of the dural sinus.^20^ The dural venous sinuses are large venous conduits within the dura mater layer of the meninges that play an indispensable role in draining venous blood from the cranial cavity.^21^ Veins are compliant, owing to the relatively low presence of elastic and muscular fibers in the venous tunica media. Thus, the elastic tissue and smooth muscle in veins are not as developed as in arteries, and hence veins are more susceptible to external compression.^22^ Extrinsic stenosis may initially result from a small increase in ICP, which leads to a greater increase in ICP. This positive feedback loop is termed the Starling‐like resistor (Fig. 2). This model proposes that the external pressure of the box surrounding the tube, which depends on the flow rate, can affect the diameter of the flow area. Therefore, an initial moderate elevation in ICP may result from factors such as weight gain and hormonal changes. Intracranial hypertension compresses the collapsible transverse sinus, causing venous outflow obstruction, resulting in reduced CSF absorption into the venous sinus and consequently further elevation in ICP, thus perpetuating the entire cycle. Eliminating the effects of sinus stenosis through venous stenting and other treatments can theoretically end this vicious circle.^23^, ^24^

#### DEGREE OF STENOSIS

The degree of stenosis as assessed by magnetic resonance venography (MRV) was graded in a prior study of 51 patients with IIH and venous sinus stenosis.^25^ This study found no correlation between CSF opening pressure and the degree of venous sinus stenosis, which indicates that there is a compensatory mechanism of collateral channel opening for greater stenosis. In addition, a recent study demonstrated a lack of correlation between the degree of stenosis and clinical presentation such as visual prognosis.^26^ Therefore, the degree of stenosis observed on MRV should not be the only determinant of stent placement or diagnostic of IIH. In conclusion, no association has been observed between the degree of anatomically defined stenosis and the hemodynamics of the stenosis.

#### CAUSE vs. CONSEQUENCE

ICP can be reduced through effective treatment, such as CSF removal or diversion, thus proving the reversibility of stenosis and suggesting that venous sinus stenosis is a consequence of the pathological process of IIH rather than a causal agent.^27^ However, chronic venous sinus compression may lead to local fibrosis and remodeling of the sinus wall, producing inherent stenosis and ultimately elevating ICP.^28^ Therefore, we consider that venous sinus stenosis and IIH tend to show a bidirectional cause‐effect relationship.

#### INTRINSIC vs. EXTRINSIC STENOSIS

Intrinsic stenosis is anatomically a local filling defect caused by a relatively fixed internal structure of the lumen that can alter venous flow dynamics, including the presence of AGs, organized chronic thrombosis, and embryonic remnants.^16^ Studies have shown that 70% of patients with unilateral venous sinus stenosis have AGs, while only 18% of patients with no transverse sinus stenosis have AGs.^29^ AGs are invaginations of the arachnoid membrane that protrude into the dural venous sinuses through the gaps of the dura mater, thus providing a pathway of CSF resorption from the subarachnoid space into the bloodstream.^30^ Such normal anatomical variations exist in most venous sinuses, but some authors believe that a subset of patients have predisposition toward exhibiting increased ICP, which may act as a primary mediator of IIH pathophysiology.^31^ AGs can cause asymptomatic stenosis due to intrinsic outflow restriction, causing individuals to be vulnerable to symptomatic lesions when ICP increases. For example, in nonobese patients, when ICP and venous outflow are normal, these anatomical variations do not obstruct CSF drainage. However, they may lower the pathological threshold in obese patients with IIH. It should be noted that as ICP increases in obese patients, anatomical variations may prevent an adequate compensation of the venous sinuses.^32^

**FIGURE 3:**
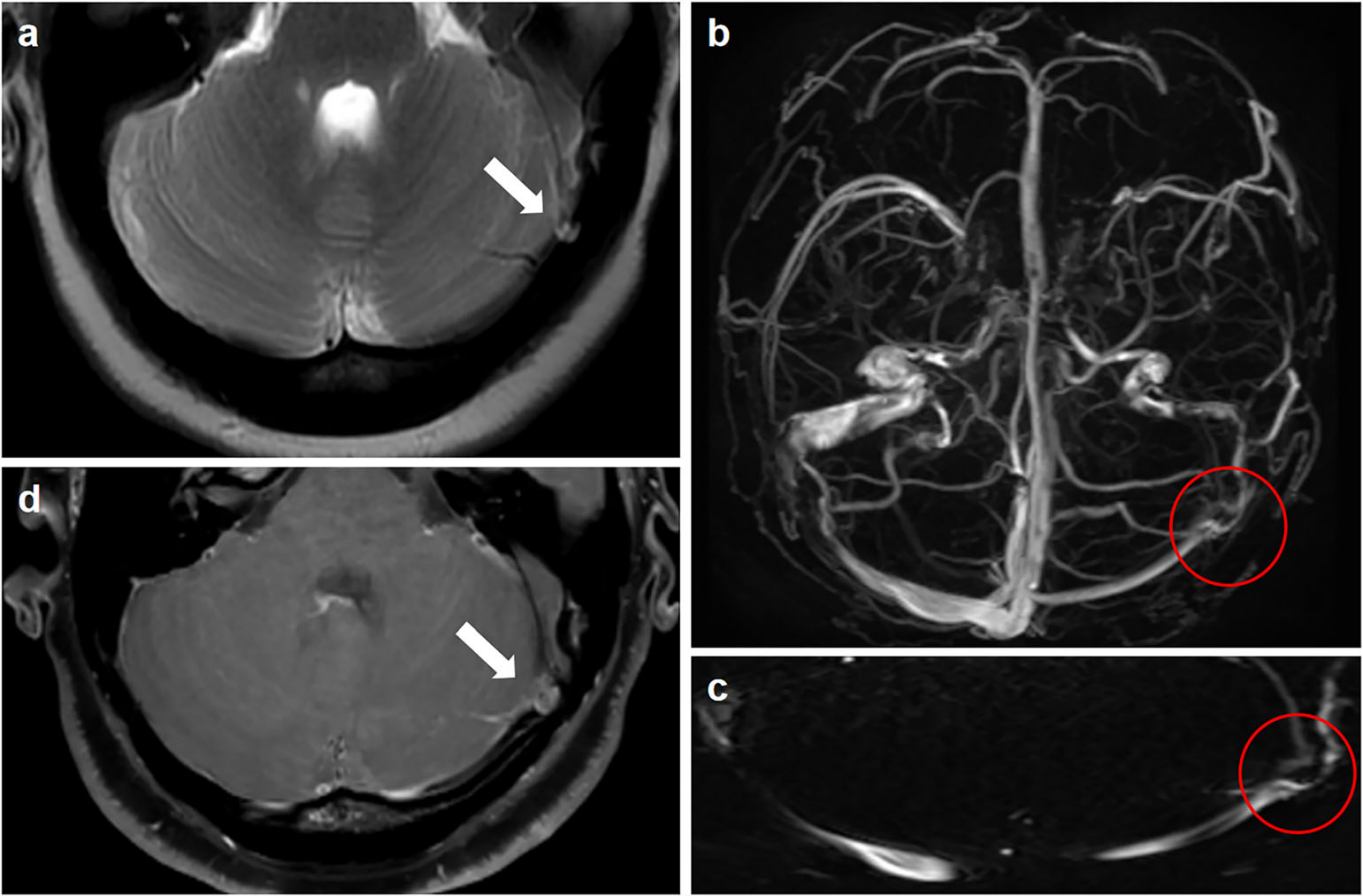
Classical signs suggestive of intrinsic stenosis. (**a**) Axial T2‐weighted magnetic resonance imaging (MRI) showing an arachnoid granulation (arrow). (**b**) Inferior reconstruction of magnetic resonance venography (MRV) demonstrating intrinsic transverse venous sinus stenosis from an arachnoid granulation (red circle). (**c**) Curved reformat of the dural venous sinus from the MRV showing that intrinsic stenosis may appear as discontinuous points of stenosis from an arachnoid granulation (red circle). (**d**) High‐resolution 3D T1‐weighted SPACE showing unilateral sinus stenosis caused by the protrusion of an arachnoid granulation into the lumen of the sinus (arrow).

Previous studies have shown that AGs are formed as ICP increases, which may form a positive feedback mechanism.^33^ In patients with intrinsic stenosis, the permeability of the junction between the perivenous CSF space and venous sinus is impaired and may lead to the reactive growth of AGs. The appearance of IIH may be slightly delayed initially due to an increase in the exchange area between the CSF and the venous system as well as the compensatory ability of the lymphatic outflow pathway. However, AGs may eventually cause mechanical obstruction of the venous sinuses as AGs increase, thus resulting in pressure elevation in the dural sinus and reduction in the efficiency of the venous CSF outflow pathway (Fig. 3).^17^

**FIGURE 4:**
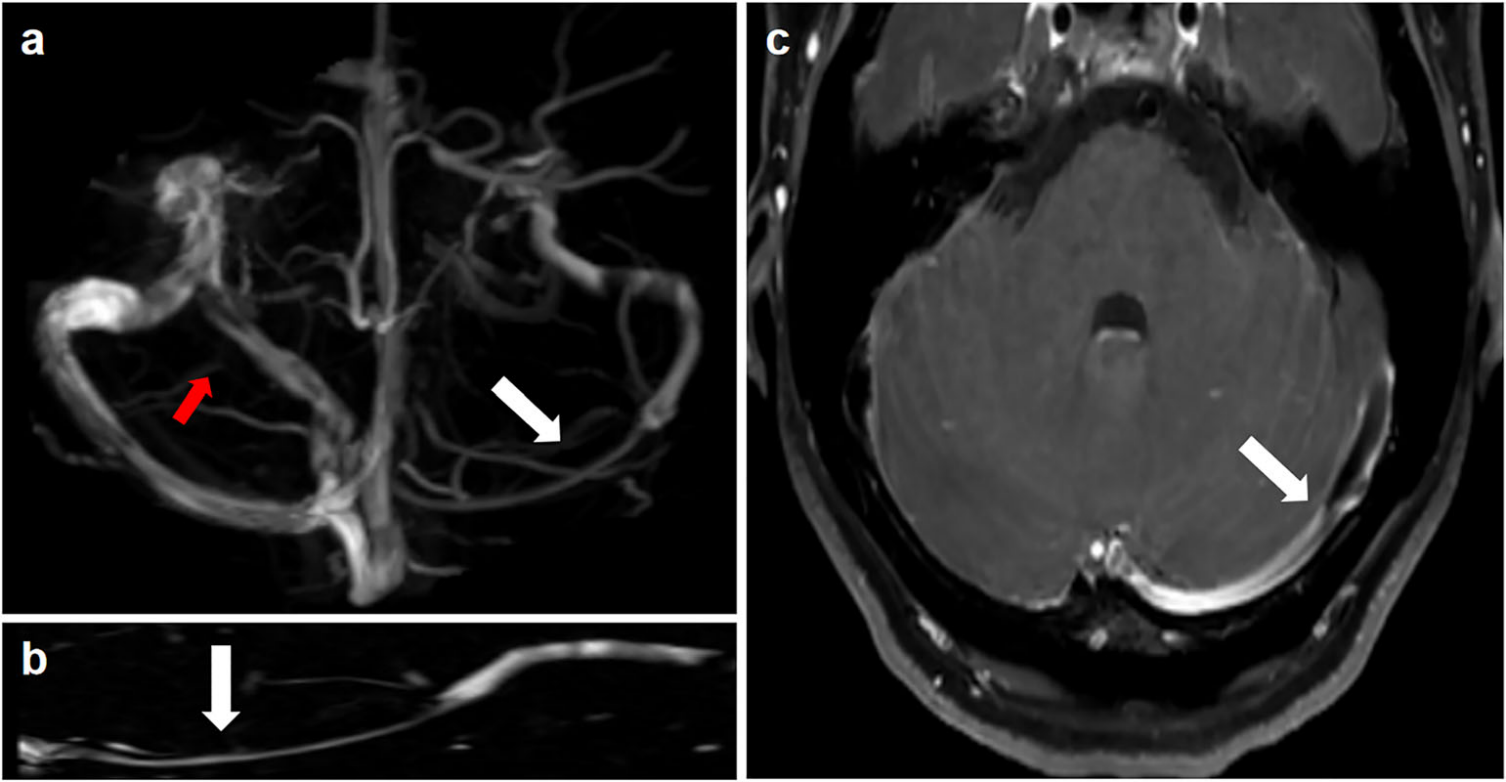
Classical signs suggestive of extrinsic stenosis. (**a**) Inferior reconstruction of magnetic resonance venography (MRV) demonstrating unilateral transverse venous sinus stenosis (white arrow) and a compensatory mechanism of collateral channel opening for this greater stenosis (red arrow). (**b**) Curved reformat of the dural venous sinus from the MRV showing that extrinsic stenosis is usually continuous in length (arrow). (**c**) High‐resolution 3D T1‐weighted SPACE showing compression of the transverse sinus by the adjacent brain parenchyma (arrow).

Extrinsic stenosis is defined as the external compression of the venous sinuses by the adjacent brain parenchyma. Transverse venous sinuses are susceptible to extrinsic stenosis because of their potential to collapse in the setting of increased ICP.^16^ In patients with extrinsic stenosis, the lymphatic outflow pathway may not sufficiently compensate for the primary damage to the venous outflow pathway. The increased volume of the CSF causes the venous sinuses to be compressed against the skull, thus resulting in extrinsic transverse venous sinus stenosis. The original stenosis fades with the decrease in ICP after CSF removal.^17^ Hence, extrinsic stenosis is not considered an independent cause of IIH. Sundararajan et al^34^ demonstrated that most patients with IIH have extrinsic stenosis. They regarded the following situation as a prerequisite: intrinsic stenosis preceding segments of extrinsic stenosis and then overall stenosis categorized as extrinsic stenosis. Among 115 patients with IIH, 75 (65%) had extrinsic stenosis originating from the overlying brain parenchyma extending from the transverse sinus. Among these patients, 17 had dominant extrinsic stenosis, obvious AGs were superimposed on the side and showed a compensatory enlargement to promote further reabsorption of CSF,^35^ while the remaining 58 cases presented with extrinsic stenosis only. Forty patients (35%) had intrinsic stenosis associated with either one or a cluster of prominent AGs located at the junction of the transverse sinus (Fig. 4).

From the above discussion, we believe that extrinsic stenosis is not an independent factor in IIH. Moreover, a higher proportion of patients with extrinsic stenosis were found in the cohort study conducted by Sundararajan et al, in which extrinsic stenosis occurring after intrinsic stenosis was attributed to external causes. Extrinsic stenosis is usually continuous. In contrast, intrinsic stenosis may appear as discontinuous points of stenosis. Patients with IIH may have characteristics of both intrinsic and extrinsic stenosis. In conclusion, whether stenosis is extrinsic or intrinsic may be only one factor that interweaves with other factors in leading to the development of IIH. This investigation will help us understand the underlying pathological mechanism of this disease more deeply.

## Imaging Findings

Imaging has played an increasingly important role in the diagnosis of IIH. For patients with suspected increased ICP, the use of MRI and MRV to exclude secondary causes and to detect subtle changes has become increasingly common. In addition, various orbital findings, empty sella, meningocele, and transverse venous sinuses are positive neuroimaging findings often observed in IIH.^36^

**FIGURE 5:**
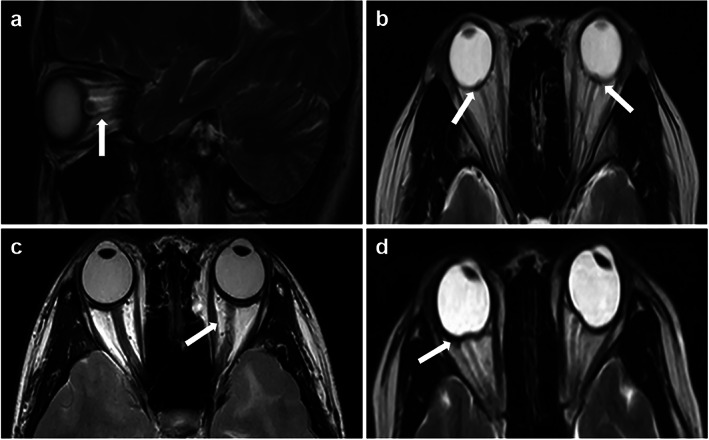
Classical magnetic resonance imaging (MRI) signs suggestive of orbital findings with idiopathic intracranial hypertension (IIH). (**a**) Sagittal T2‐weighted MRI showing dilation of optic nerve sheath (arrow). (**b**) Axial T2‐weighted MRI showing a swollen optic nerve head (arrows). (**c**) Axial T2‐weighted MRI showing tortuosity of the optic nerves (arrow). (**d**) Axial T2‐weighted MRI showing posterior globe flattening (arrow).

When ICP rises, the resulting changes in pressure can be transmitted to the subarachnoid space in the optic nerve sheath and the accumulation of CSF can lead to the expansion of the optic nerve sheath.^37^ Dilatation of the optic nerve sheath can be reliably detected by MRI. The sensitivity and specificity of MRI for diagnosing optic nerve sheath dilatation in patients with IIH were 72%–80% and 96%, respectively.^38^ Optic nerve protrusion, an MRI manifestation of papilledema due to increased pressure of optic nerve sheath CSF, is a clinically relevant marker of papilledema risk in IIH.^39^ This intraocular protrusion manifests as a focal hyperintensity at the optic nerve head.^40^ The elevated CSF pressure can distort the intraorbital optic nerve due to the fixation of the nerve at the proximal and distal points. Detection of tortuosity depends on the MRI slice thickness and orientation.^41^ The balance between ICP and intraocular pressure maintains the normal convexity of the posterior globe. The elevated CSF pressure also straightens the curvature of the posterior sclera attached to the optic nerve, which is known as scleral flattening (Fig. 5).^42^

**FIGURE 6:**
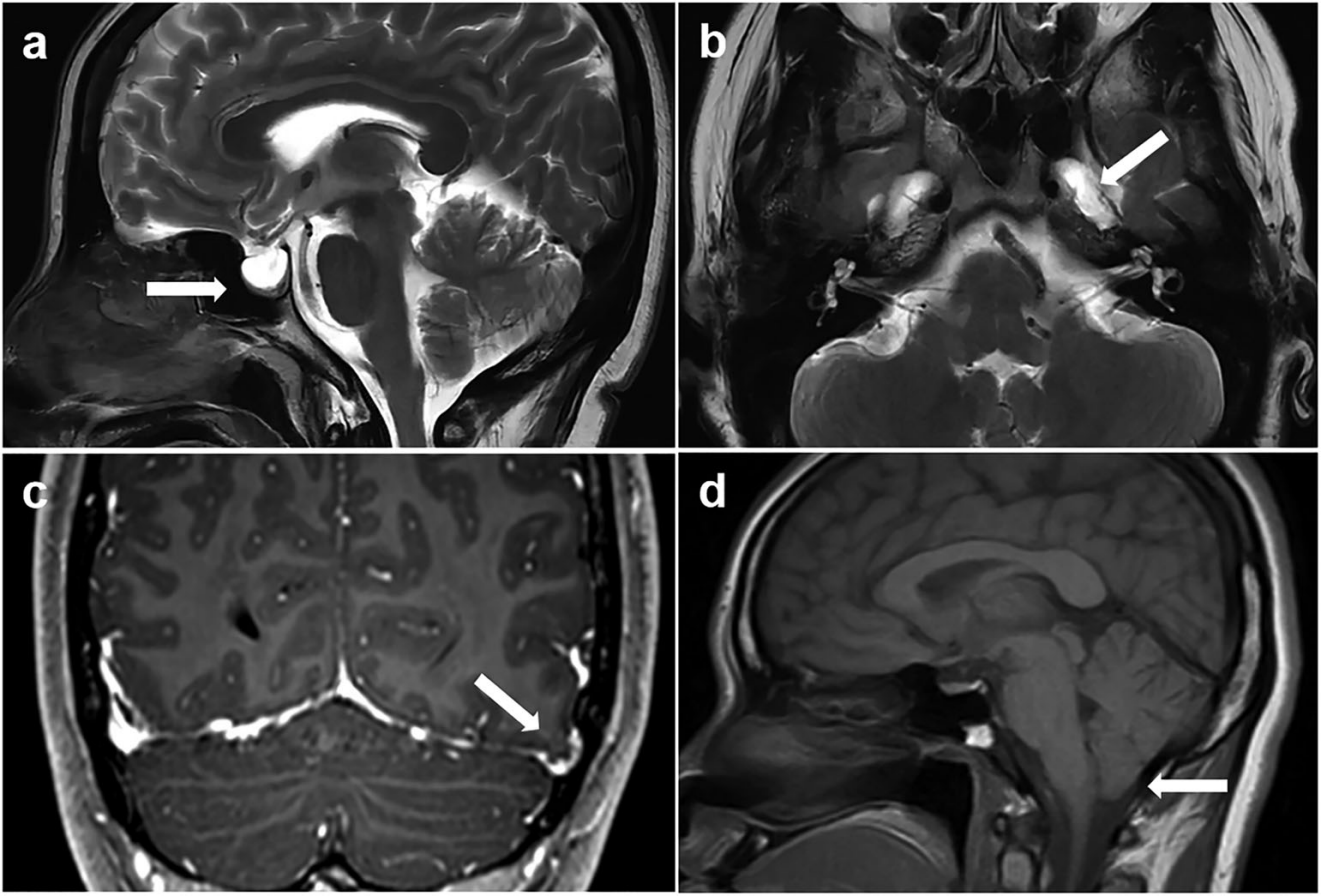
Classical magnetic resonance imaging (MRI) signs suggestive of elevated intracranial pressure. (**a**) Sagittal T2‐weighted MRI showing an empty sella (arrow). (**b**) Axial T2‐weighted MRI showing the enlarged Meckel's caves bilaterally (white arrow points to left Meckel's cave). (**c**) Coronal T1‐weighted postcontrast MRI showing a left temporal lobe cephalocele (arrow). (**d**) Sagittal T1‐weighted MRI showing mild inferior cerebellar tonsillar descent (arrow).

Despite the constant pulsation of CSF in the setting of elevated ICP, the CSF of the subarachnoid space can herniate through the infundibular hiatus of the diaphragm sella into the sella, and the pituitary gland is compressed against the floor of the sella, which corresponds to a neuroimaging finding called “empty sella.” This symptom is commonly seen in older patients due to developmental defects in the diaphragm sella. When young women present with the empty sella, especially those with clinical symptoms such as headaches, IIH should be strongly suspected.^42^ Patterson et al^43^ found that an MRI‐measured pituitary‐to‐sella turcica ratio of less than 0.5 increased the likelihood of increased ICP.^44^ The pathophysiological manifestations of the sellar area are similar to those of various skull base defects such as meningocele and encephalocele. Up to 10% of patients with IIH may have enlarged Meckel cavity and obvious meningocele; however, these findings are rarely observed in normal controls.^45^ In addition, cerebellar tonsillar ectopia as an imaging finding may be confused with other etiologies. It must be emphasized that the abovementioned imaging findings can only indicate the lack of correlation between the sensitivity of imaging findings and the prevalence of IIH. In other words, the diagnosis of elevated ICP cannot be ruled out without the above findings (Fig. 6).^30^

Venous sinus stenosis is the most sensitive and reliable diagnostic indicator for the presence of IIH, in contrast to the abovementioned neuroimaging signs.^20^ Even in cases with few clinical manifestations, the presence of venous sinus stenosis emphasizes the importance of early diagnosis in preventing progression to irreversible vision loss.^46^ Therefore, we conclude that venous sinus stenosis plays a key role in the study of the pathophysiological mechanisms of IIH. This is the reason for the special focus on this putative mechanism within the current review.

**FIGURE 7:**
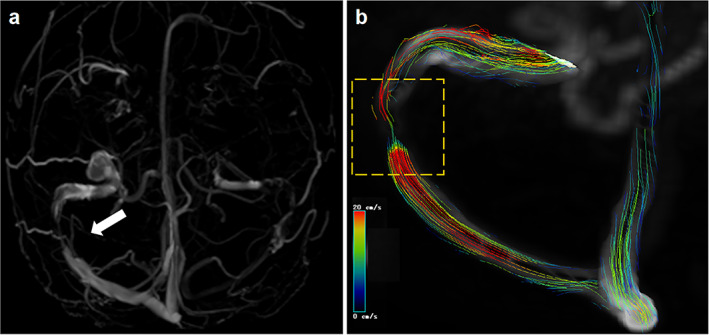
Four‐dimensional (4D) flow magnetic resonance imaging (MRI) showing the hemodynamic of cerebral venous sinus stenosis. A female presented with severe headache and papilloedema with a clinical diagnosis of idiopathic intracranial hypertension (IIH). (**a**) Inferior reconstruction of magnetic resonance venography (MRV) showed the drainage dominance of right transverse sinus and right transverse sinus stenosis. (**b**) 4D flow pathlines indicate abnormal flow condition and the existence of stenosis. The color coding of the pathlines reflects the magnitude of blood flow velocities in the vasculature (blue = low, red = high). The white arrow and yellow‐dashed square in the artwork mean the transverse sinus stenosis.

In recent years, emerging imaging technologies have been introduced in the field. Three‐dimensional (3D) T1‐weighted SPACE (Sampling Perfection with Application Optimized Contrast using Different Angle Evolutions) imaging has proved helpful in diagnosing various intracranial venous system diseases.^47^ This 3D fast spin echo black blood sequence can directly show the venous vessel wall, thus demonstrating the degree of stenosis and distinguishing between intrinsic and extrinsic stenosis. In addition, four‐dimensional flow MR imaging is a time‐resolved, 3D velocity‐encoded MR imaging technology that allows for the acquisition of dynamic, multidirectional data on blood flow and has recently been used to evaluate intracranial venous flow (Fig. 7).^48^ These methods may be particularly useful for unveiling the mechanisms and imaging findings involved in IIH as well as for therapeutic purposes.

## Management of IIH

IIH is a disease that produces symptoms and signs of ICP without an alternative cause. The main goals of treatment are to protect visual function and alleviate symptoms.^49^ The latest treatment guidelines can be summarized as treating underlying diseases, protecting vision, and minimizing headache morbidity. Weight loss is the cornerstone of treatment; acetazolamide can inhibit carbonic anhydrase, thereby reducing the production of CSF by the choroid plexus and surgery is required when there is a rapid or progressive decline in visual function. Surgical treatments include CSF shunting (ventricular‐peritoneal shunt and lumbar‐peritoneal shunt), optic nerve sheath fenestration (ONSF), and venous sinus stenting.^1^, ^50^

**FIGURE 8:**
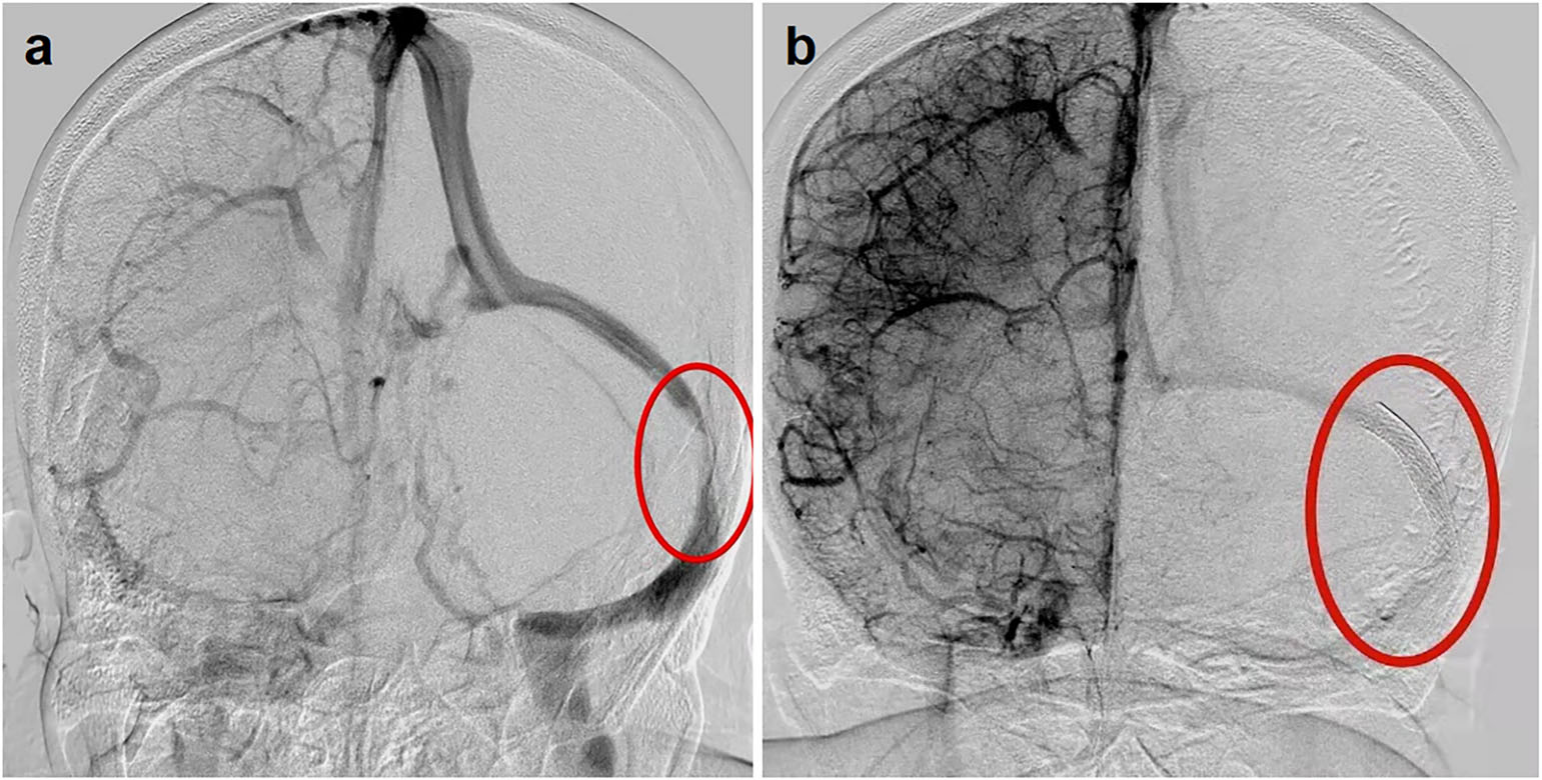
Venous sinus stenting in patients with idiopathic intracranial hypertension (IIH). A patient with IIH demonstrating severe left‐sided transverse‐sigmoid stenosis (**a**). After stent placement, the normal caliber of the sinus is restored (**b**). The red circle (a) means the transverse‐sigmoid stenosis; The red circle (b) means the venous sinus stenting.

CSF shunts are suitable for vision loss, papilledema, and obvious systemic symptoms of increased ICP. ONSF is the first‐choice treatment for patients with vision loss caused by severe papilledema without obvious headache.^51^ However, these two methods have high postoperative risk and recurrence rates. A 2015 meta‐analysis compared venous sinus stenting, CSF shunts, and ONSF in the treatment of IIH. Venous sinus stenting, which has emerged in recent years, was found to be more effective and involve fewer complications than the other two options.^52^ Therefore, venous sinus stenting is a more suitable therapeutic intervention for IIH (Fig. 8).

A study by Nicholson et al^53^ showed that recurrence seems to preferentially affect young obese female patients with extrinsic stenosis and high CSF opening pressure after venous sinus stenting. Moreover, Kumpe et al^54^ showed that the recurrence rate of the intracranial venous pressure gradient was significantly higher in patients with extrinsic stenosis than in patients with intrinsic stenosis. The above conclusions support the pathophysiological hypothesis of extrinsic stenosis formation and prove that these stenoses are caused by extrinsic compression of the venous sinuses. Based on the above results, current medical guidelines recommend choosing the length of the stent according to the type of stenosis. Patients with extrinsic stenosis may need one or two long stents to cover the transverse sinus from the torcula to the sigmoid sinus, while a single shorter stent usually seems to be sufficient to treat patients with intrinsic stenosis. The key to this technology may be in reducing the recurrence rate.^54^, ^55^ At the same time, the intracranial venous sinuses are dynamic structures. Although patients with IIH often have bilateral venous sinus stenosis, a stent on only one side may be sufficient to mitigate the symptoms.^51^, ^56^ Patsalides et al^31^ conducted follow‐up angiography and venous pressure measurement studies on a group of patients after stent placement and observed a (not statistically significant) trend toward a greater reduction in ICP within the intrinsic stenosis group. This observation has not been confirmed, but it may reflect the fact that either extrinsic or intrinsic stenosis may be only one of the factors that interacts with other factors to result in IIH. This also confirms our analysis of the relationship between venous sinus stenosis and IIH.

## Conclusion

IIH was first discovered more than 100 years ago, and the past decade has seen improvements in the understanding and diagnosis of IIH. The prevalence of this condition is expected to increase as the global obesity burden continues to escalate. Therefore, further studies are needed to elucidate the pathophysiology and underlying mechanisms of IIH.^57^ Over the years, imaging has played an increasingly important role in the diagnosis and treatment of IIH. Venous sinus stenosis is the most important imaging manifestation and is an important component in the mechanisms of IIH. To date, no correlation has been observed between the degree of venous sinus stenosis and the degree of ICP. Therefore, it is necessary to use venous manometry (which is the gold standard measure) to determine whether there is a venous pressure gradient on the stenosis and manage IIH. Even though the latest study showed that the proportion of extrinsic stenosis in IIH is relatively higher than that of intrinsic stenosis, the extrinsic stenosis is relieved by decrease in ICP, which may be regarded as a nonindependent factor. Therefore, we believe that the occurrence of IIH is a result of the intertwining of various factors. Although our understanding of the relationship between venous sinus stenosis and IIH has increased, there is still much to be known, and the pathophysiology of this condition needs further exploration.
